# Dentistry as a prospective career choice in india

**DOI:** 10.6026/973206300161134

**Published:** 2020-12-31

**Authors:** RN Arun Kishore, K Yuvaraj Babu, R Gayatri Devi

**Affiliations:** 1 Saveetha Dental College, Saveetha Institute of Medical and Technical Sciences, Saveetha University, Chennai 77, India

**Keywords:** Career choice, dentistry, dental survey, career in dentistry

## Abstract

Career choice is a complex decision in a student's life. The opportunity to participate in dental education in many countries, especially in the developing ones, is limited to a small percentage of the community. There is a wide range of options for students to choose as a career in general family, gender, personal interest; outcome expectancies can affect the decision in choosing it. Many studies showed that many individuals find themselves in occupations not really knowing why they made that particular decision. The changing nature of the dental workforce and the need to retain the services of future members has made it important to understand why current dental students have chosen dentistry as their career. However, the choice of dentistry becomes forceful at times by peer pressure, cultural thrust or inability to procure medicine. It is of interest to evaluate dentistry as a prospective career choice in India. The participants answered a questionnaire based online survey and the results were collected and analysed statistically. Analyses of data from the survey shows that majority (36.72%) of students had chosen dentistry as a career choice having missed entry or selection into medicine.

## Background:

The choice of career is a very complex decision made by a student since it decides the kind of profession they choose to pursue in their life [1]. The choice of a career is a critical decision that has an obvious impact on future life patterns [2]. Dentistry is one among the best professions in the present day. Dentistry occupies an important position in society as licensed healthcare workers [3]. It is important to understand the priorities and the background of those choosing dentistry [4]. Some make decisions by taking the path chosen by parents or following the steps of the elder sibling [5] technology has been developing day to day which is highly useful in educational aspects for students. There is a wide range of options for students to choose as a career, in general family, gender, personal interest; outcome expectancies can affect the decision in choosing it [1]. Many studies showed that many individuals find themselves in occupations not really knowing why they made that particular decision [2]. Many issues may be considered when choosing a career profession even gender and ethnicity may also influence the decision [4] It varies from person to person and then personal characteristics and motives of the students also plays a major role in shaping their career performance [6]. Choosing a career is one of the most important decision students ever make [7 - check with author]. Over the past years various research done by our team was on Osteology [8-11] stature estimation, [12] uses and ill effects of electronic gadgets [13,14], on RNA [15,16] animal studies [17] and in few other fields [18,19]. Therefore, it is of interest to evaluate dentistry as a prospective career choice in India.

## Materials and Method:

A prospective descriptive study was done through google docs, for this survey a self-structured questionnaire was created with 15 questions and 128 subjects. The survey was approved by SRB of the Institution. The sampling method used was simple random sampling. Minimizing the errors in the questioning, planning the questions in simple language and avoiding leading questions were the steps taken to reduce the bias. The responses obtained were analysed using Chi-Square analysis in SPSS software and represented by a bar chart and table.

## Result and Discussion:

From the survey the percentage of responses for various questions by the participants are tabulated in Table 1 (see PDF). In the present study we had noticed that the majority of the students 36.27% has chosen dentistry, as they did not get medicine closely followed by peer influence, which was opted by 32.81% of participants. Over 59.38% of participants did not like dentistry and around 42.19% of participants would not recommend dentistry as a career for others. The participants were divided equally for the question if dentistry was better than medicine. Among the participants only 35.16% accepted that dentist was treated similar to medical doctors whereas 50% has opted no for this question. 54.69% believe the reason for it to be having less knowledge than medical doctors and 29.69% feel they are educated less than them. Only 16.41% opted that a dentist earns more than a doctor 54.69% students opted no for it. For the question why they opted for dentistry35.94% opted flexible life style, 21.09% opted for social status and to shape future of dentistry and only 7.81% opted good salary. In our survey on how to succeed in dentistry 33.59% students selected presence of mind followed by good practice, good communication and ethics & morality, while 10.94% opted all the above to succeed. Only 46.1% has opted yes for dentistry to have good future but for the question is dentistry a depressing job 54.69% opted no, 16.41% yes and 28.91% opted may be for the question. Figure 1(see PDF) shows the bar chart of association among gender for choosing dentistry as a career, on Chi square analysis no significant relationship was found between gender and various reasons for choosing dentistry as a career. When compared with previous study, self-interest was followed by inability to get admission in medicine in our present study participants opted not able to get admission in medicine and peer influence as the preferred options [1] In another study on recommending dentistry 30.6 % opted yes, which is almost similar to our study in which 29.69% opted yes [7 check with author]. In a study, why recommend dentistry 81.74% opted good future scope in a study which is much higher than 46.1% obtained in our study that is almost similar to another study where 49.22% of the participants opted good future scope [7 check with author][20]. The limitations of the study was that it was done among a small homogeneous population In future this study can be done in depth in a large heterogeneous population.

## Conclusion:

We document the various reasons for choosing dentistry as a career option in India among first-year undergraduate dental students, from this study we found most of the participants had chosen dentistry as a career having missed entry or selection into medicine.

## Funding support:

The authors declare no funding support for this study.
